# Caregiving and Financial Hardship Among Middle-Aged and Older Adults

**DOI:** 10.1093/geroni/igaf122.2329

**Published:** 2025-12-31

**Authors:** Seokmin Kim, Kingsley Mbam

**Affiliations:** University of Massachusetts Boston, Quincy, Massachusetts, United States; University of Massachusetts Boston, Boston, Massachusetts, United States

## Abstract

Studies have shown a negative association between caregiving and financial hardship. However, relatively few studies examined this association among middle-aged and older individuals, despite the increasing age of caregivers. Using data from the 2018 Health and Retirement Study, this study investigated the financial impact of caregiving in middle adulthood and later life. The respondents include individuals aged 51 years and older (n = 4569). Caregiving is assessed as a binary measure: 1= provided care at least once a month, 0 = did not provide care in the last month. Financial hardship is assessed utilizing four hardship indicators - difficulty paying bills, ongoing financial stress, medication reduction due to cost, and food insecurity. Respondents are considered to be in financial hardship if they experienced two or more of the four hardship indicators. Results from logistic regression models showed that providing care in middle and old age is associated with a substantially higher likelihood of experiencing financial hardship (OR: 1.36; 95% CI: 1.14-1.62). These findings call for policy interventions to help middle-aged and older caregivers maintain financial stability and security. Out-of-pocket cost reimbursement programs can be developed to reimburse caregivers for expenses incurred in their provision of care.

